# Tissue Engineering: Understanding the Role of Biomaterials and Biophysical Forces on Cell Functionality Through Computational and Structural Biotechnology Analytical Methods

**DOI:** 10.1016/j.csbj.2019.04.008

**Published:** 2019-04-17

**Authors:** Nour Almouemen, Helena M. Kelly, Cian O'Leary

**Affiliations:** a School of Pharmacy, Royal College of Surgeons in Ireland, 123 St. Stephen's Green, Dublin 2, Ireland.; b Tissue Engineering Research Group, Dept. of Anatomy, Royal College of Surgeons in Ireland, 123 St. Stephen's Green, Dublin 2, Ireland.; c Advanced Materials and Bioengineering Research (AMBER) Centre, Royal College of Surgeons in Ireland and Trinity College Dublin, Dublin 2, Ireland.

**Keywords:** Tissue engineering, Biomaterials, Polymer, Mechanobiology, Mechanotransduction

## Abstract

Within the past 25 years, tissue engineering (TE) has grown enormously as a science and as an industry. Although classically concerned with the recapitulation of tissue and organ formation in our body for regenerative medicine, the evolution of TE research is intertwined with progress in other fields through the examination of cell function and behaviour in isolated biomimetic microenvironments. As such, TE applications now extend beyond the field of tissue regeneration research, operating as a platform for modifiable, physiologically-representative *in vitro* models with the potential to improve the translation of novel therapeutics into the clinic through a more informed understanding of the relevant molecular biology, structural biology, anatomy, and physiology. By virtue of their biomimicry, TE constructs incorporate features of extracellular macrostructure, molecular adhesive moieties, and biomechanical properties, converging with computational and structural biotechnology advances. Accordingly, this mini-review serves to contextualise TE for the computational and structural biotechnology reader and provides an outlook on how the disciplines overlap with respect to relevant advanced analytical applications.

## Graphical Abstract

**Figure:**
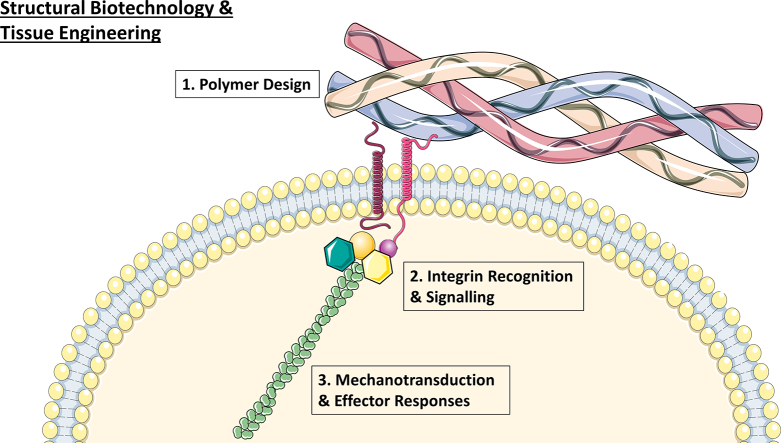

## 1 Introduction

Since the seminal paper of Langer and Vacanti over 25 years ago [1], tissue engineering (TE) has grown enormously as a science and as an industry. In 1993, it was introduced to the wider scientific community as “an interdisciplinary field that applies the principles of engineering and the life sciences toward the development of biological substitutes that restore, maintain, or improve tissue function”. As an industry, TE has stabilised and become profitable since the turn of this decade [2]; within Europe, for example, more TE products are regularly emerging in registered clinical trials as advanced therapeutic medicinal products (ATMPs) [3]. Classically, TE recapitulates tissue and organ formation in our body to varying degrees, bringing together cells in a three-dimensional (3D) fabricated environment where appropriate signals are provided for tissue formation. In parallel, the evolution of TE research is intertwined with progress in other fields through the examination of cell function and behaviour in isolated biomimetic microenvironments. Expanding our knowledge of stem cell biology [4,5], disease [6,7], and improved maintenance of cells in culture without dedifferentiation [8,9]. As such, TE applications now extend beyond regeneration strategies alone, operating as a platform for modifiable, physiologically-representative *in vitro* models [10]. Indeed, studies with TE *in vitro* models can answer questions that potentially improve the translation of novel therapeutics and ATMPs from the laboratory bench into the clinic, through a more informed understanding of the relevant molecular biology, structural biology, anatomy, and physiology.

**Fig. 1:**
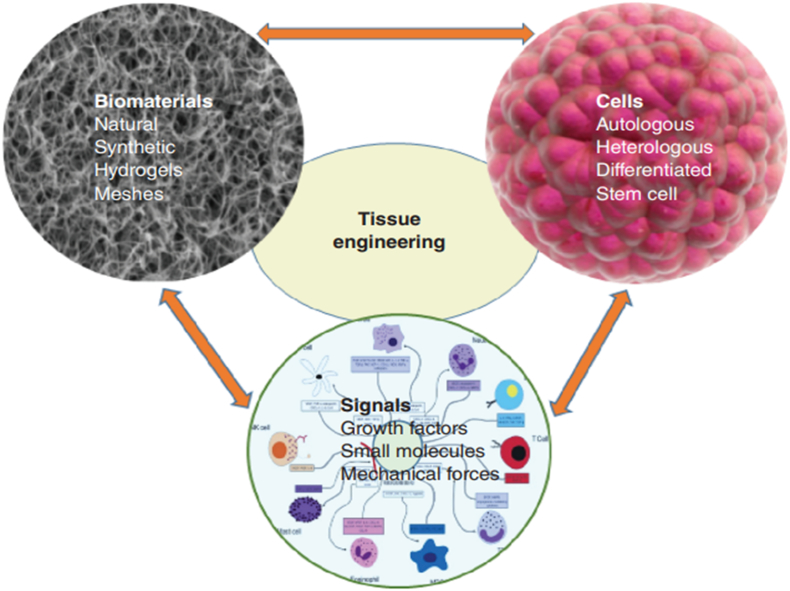
The tissue engineering triad. A combination of cells cultured on a biomaterial scaffold with appropriate biophysical and chemical signals coordinate to recapitulate the desired tissue. Image adapted from [11].

The manufacture of a successful TE construct is underpinned by three crucial components, referred to as the tissue engineering triad (Fig. 1): a relevant selection of cells, a biomaterial scaffold for 3D culture, and the presence of appropriate signals such as biophysical cues and chemical mediators that coordinate to ultimately recreate tissue [11]. Scaffolds produced should be biocompatible to preclude an immune response in the host following implantation, maintain a biodegradability rate that facilitates the replacement of scaffold with physiological tissue without collapse of the construct, have mechanical properties that mimic that of its surrounding *in vivo* environment, an architecture that facilitates cellular processes like diffusion, vascularisation and waste removal, and finally, be feasible in efficient and economic manufacture [12]. Signals to encourage extracellular matrix (ECM) production by cells can be provided by biophysical cues such as those applied by a bioreactor in which the construct is cultured [13], by delivery of bioactive molecules or genes [14,15], or even by the cell substrate's biophysical properties [16]. Cell sources include stem cells and host-derived cells that are typically cultured *ex vivo* on the biomaterial before implantation [17].

As the field of TE has evolved, an increased convergence with computational and structural biotechnology research has occurred as a function of the former's inherent biomimicry. Conventionally, structural biotechnology is primarily concerned with nanoscale molecules and how they interact within a biological system, such as inside a cell or tissue [18]. The ability to investigate the interaction between complex macromolecules like proteins and nucleic acids has been enabled on an unprecedented scale by advances in imaging, informatics, and big data omics, permitting analytical outputs of biological function and disease that were hitherto impossible or unfeasible [19,20]. On the other hand, the discipline of TE conventionally explores the “macro” level, creating constructs on the scale of the tissue or organ with anatomical architecture and robust bulk physical properties. However, as more knowledge has been accrued from the life sciences and as manufacture technologies have advanced to the high-resolution fabrication techniques of electrospinning and 3D printing additive manufacture [[21], [22], [23]], TE constructs can now accurately recapitulate elements of an *in vivo* microenvironment such as extracellular macrostructure, molecular adhesive moieties, and biomechanical properties [24]. Taken together, structural biotechnology and TE thus converge in a reciprocal fashion, whereby TE model and construct design provide the inputs to elicit certain cell and tissue functions, while the outputs that are stimulated can be identified, quantified, and interpreted by structural biotechnology approaches. Indeed, techniques such as high resolution microscopy and next generation sequencing, coupled with bioinformatics, can provide a deeper analysis of TE platforms and are being increasingly recognised within the field as powerful methods for extensive analysis of *in vitro* 3D models [25].However, the respective core concepts, strengths, and opportunity for synergy are not yet widely recognised or understood across both fields, prompting the need to create some context that can act as a springboard for further interest and collaboration among disciplines.

Accordingly, this mini-review serves to contextualise TE for the computational and structural biotechnology reader and provides an outlook on how the disciplines overlap with respect to relevant advanced analytical applications. While cell sources are a critical consideration within TE platforms, this review considers the structural biologist to have extensive experience in cellular biology. Therefore, we focus on the role of extracellular polymeric structures and the fundamentals of how their properties can transduce intracellular signals as integral facets to the biomaterial and signal pillars of the TE triad.

## 2 The Use of Natural Polymers in Tissue Engineering as Biomaterials

**Fig. 2:**
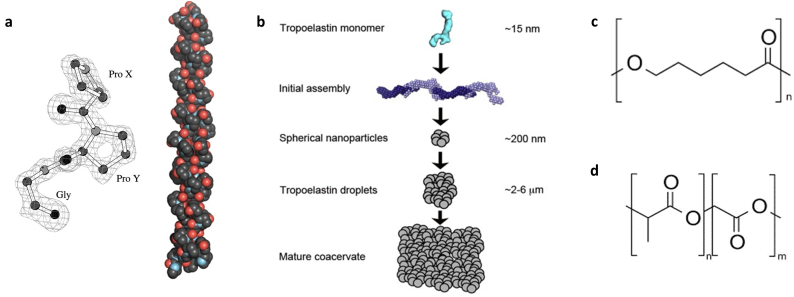
(a) Collagen structure, composed of repeating Gly-X-Y units that assemble into a heterotrimeric structure. Adapted from [31,32]. (b) Formation of tropoelastin coacervates in elastin synthesis. Adapted from [33]. (c) Poly-Ɛ-caprolactone (PCL). (d) Poly(lactic-*co*-glycolic) acid (PLGA) structure.

In fundamental terms, biomaterials are natural, synthetic, or composite polymer constructs that have been manufactured to a defined set of parameters in order to interact with a biological system (Fig. 2). The critical role of the biomaterial is to mimic the extracellular matrix (ECM) to which cells anchor and orientate themselves to form tissue, which itself is primarily composed of elastic fibres, collagens, and integrated glycosaminoglycans [26]. Thus, in addition to being non-toxic for the cells, biomaterials need to be structurally designed to reflect the elastic and tensile properties of these ECM components, in addition to exhibiting the capability for further crosslinking reactions that can occur *in vivo* [27]. Also, biomaterials must display cell-binding moieties for attachment, either by resembling the interactions of native ligands or by direct incorporation of peptide sequences, such as the triple helical GFOGER sequence of fibrillar collagens [28], the RGD epitope within fibronectin [29], or the GRKRK motif of tropoelastin [30]. Finally, and particularly in the case of synthetic materials, biodegradability and bioresorption are important issues to consider in order to ensure that cells have the capability to break down polymeric units and replace them with their own secreted ECM, if desired.

Of course, one of the simplest methods to mimic the ECM is to isolate and use the natural polymers that constitute it. As the most abundant structural protein *in vivo*, collagen is one of the most popular biomaterial choices in the TE field [34]. Collagens form a large protein family containing more than 40 genes encoding various alpha chains which can form at least 29 members, within which type I collagen forms the bedrock of the ECM of many tissues [35]. Collagen I is fibrillar in structure, composed of a distinctive heterotrimeric left-handed helix (Fig. 2a). The repeating Gly-X-Y amino acid sequence in its primary structure is critical to the formation of the polypeptide and resultant fibrils, reducing steric hindrance to permit helical formation and outward projection of other amino acid side chains to maximise adhesion and crosslinking. For almost every TE application, type I collagen has been explored as a biomaterial substrate, in many forms: as polymerised hydrogels [[36], [37], [38], [39]], porous polymeric scaffolds [[40], [41], [42]] and within decellularised tissue [[43], [44], [45]].

Other natural polymers can offer different mechanical or functional properties to those of collagen in biomaterials, which can be useful for particular regenerative medicine applications or TE *in vitro* disease models. For instance, while collagen confers stability and tensile strength to the ECM, other components such as elastin endow tissue with improved elastic properties [33]. Composed of monomeric units of tropoelastin that form a mature coacervate for subsequent deposition on a fibrillar network and crosslinking (Fig. 2b), the presence of hydrophobic regions within elastin's protein structure is integral for recoil following distension, whereby the exposure of non-polar residues in stretched form provoke a thermodynamically-favourable structural retraction to shield them from aqueous surroundings [46]. Accordingly, this natural polymer has been predominantly explored in TE for vascular and dermal applications, where such mechanical properties are inherently critical [[47], [48], [49], [50], [51], [52]]. As highlighted in these studies, elastin biomaterials have generally been fabricated as *co*-polymers with collagen, as well as silk fibroin, a versatile natural polymer [53]. Other natural polymers of notable interest in TE are hyaluronic acid and alginate. Neural ECM in the body contains higher concentrations of the glycosaminoglycan hyaluronic acid and its inclusion in biomaterials has been found to enhance tissue regeneration in intervertebral disc injury and reduce degeneration in retinal nervous tissue [54,55]. Other tissue where hyaluronic acid is concentrated, such as hyaline cartilage, has also benefited from its presence in TE constructs [56]. Interestingly, hyaluronic acid is also an intriguing polymer for TE *in vitro* disease models, where an upregulation of its presence can exacerbate metastasis and mortality in cancer [57]; indeed, different structural features of hyaluronic acid appear to mediate different inflammatory and healing responses *in vivo* [58], and in effect, different cell behaviours in healthy and diseased states.

Alginate, on the other hand, is derived from sea weed and is not present in the human body [59]. However, it is an interesting polymer for biomimetic TE *in vitro* models due to the ability to modulate its tensile and viscoelastic properties without resorting to crosslinking methods that can adversely affect cell-binding epitopes. Moreover, epitopes can be attached to alginate, providing further control of cell ligand density. In this way, alginate biomaterials have great utility in platforms where examination of the effects of mechanical properties on cell behaviour is sought that is independent of cell-ligand effects [60,61]. This is in stark contrast to collagen, where commonly used crosslinkers can hinder cell recognition of peptide sequences that are altered in the formation of ester and amide covalent bonds (discussed in [34]). Albeit a strength of alginate, this ligand-material decoupling highlights a major limitation of the use of other natural polymers: reduced capacity for customisation of physical and chemical structural properties, which translates into reduced capability for finely tuned control of TE systems for different applications. To address this issue, the TE field has investigated the use of synthetic biomaterials to provide a more controllable means of ECM biomimicry.

## 3 Structural Biomimicry With Synthetic and Composite Polymer Biomaterials

In general, the principal advantage of synthetic polymers in TE applications is their versatility. The control afforded over factors such as the structure of the monomeric units, the ratio of *co*-polymer structures, polymer sequence, chain length, and inter-chain facilitates a finer degree of control of biomaterial characteristics like biodegradability and mechanical properties [62] than that which can be achieved with natural polymers that have in effect, been pre-synthesised by nature, and exhibit a greater degree of structural heterogeneity [24]. Moreover, synthetic polymers can be designed to be more stable than protein-based polymers, permitting the use of manufacturing methods that use extremes of some conditions like charge and temperature; typical examples are electrospinning and melt electrospinning, respectively, for which the use of collagen solutions to prepare nanofibrous structures is doubtful [23,63]. Finally, synthetic materials can be manufactured more easily in bulk and are not a cost prohibitive as the time-consuming and onerous isolation and purification of natural polymers. However, a synthetic polymer's biocompatibility and facility for cell attachment is not necessarily as guaranteed as it can be for natural polymers. While the latter issue can be resolved through the incorporation of functional groups to conjugate cell ligands to in stoichiometric amounts [64], more extensive testing of novel synthetic polymers and their breakdown by-products could be required before their routine use becomes widespread in TE applications. Although many different types have been explored in the TE field (recently outlined in [62]), two archetypical biomaterials that illustrate these strengths and limitations of synthetic polymers are the polyesters poly-Ɛ-caprolactone (PCL; Fig. 2c) and poly(lactic-*co*-glycolic) acid (PLGA, Fig. 2d) [65,66].

PCL, a hydrophobic and semi-crystalline material, is renowned for its long *in vivo* residence time without chemical breakdown and for its viscoelastic behaviour that is similar to native tissue [[67], [68], [69], [70]]. Unlike natural polymers such as collagen or proteoglycans, the human body lacks enzymes that can cleave PCL chains, with the net result that its breakdown proceeds slowly via hydrolytic polymeric surface erosion over an average of three years [71,72]. When this information is taken together with its biomimetic material properties, PCL can be a very useful choice of biomaterial where prolonged scaffold support is warranted before robust tissue regeneration has occurred *in situ* that can bear significant mechanical loading. In this regard, PCL holds great promise for bone TE [[73], [74], [75]], tracheal TE [[76], [77], [78]], and intervertebral disc regeneration [79,80]. However, although PCL's structure confers resistance to rapid degradation, the polymer backbone also lacks an abundance of functional groups that can be modified for ligand attachment; as such, PCL has a reduced capacity for cell attachment. Thus, PCL is often employed in a composite biomaterial that compensates for cell binding, discussed below.

Of course, depending on the application, more flexible or rapid degradation rates might be desired for a biomaterial. In this regard, PLGA is another versatile, biocompatible, aliphatic polymer that has been widely used for drug delivery and TE applications with such properties [81]. PLGA also degrades by hydrolysis on hydrated surfaces, but unlike PCL, it degrades more rapidly because the glycolic acid monomers are more hydrophilic in nature and therefore interact more readily with water in the degradation process. Conversely, lactic acid does not degrade as rapidly as a result of its hydrophobic character. Thus, through fabricating polymers with different ratios of monomeric units present, a faster or slower degradation rate can be built into the polymer, purely as a result of its primary structure. Additionally, different molecular weights can also be utilised to control biodegradation, with slower degradation as molecular weight increases. Following hydrolysis, however, the build-up of acidic breakdown products can be damaging for surrounding local tissue, with is particularly undesired in cases of tissue regeneration [82]. For TE applications where these products can be readily removed, such as in 3D *in vitro* models that have regular media changes, cellular damage and toxicity might not be as significant. In summary, taking the advantages and disadvantages of both synthetic polymers together, synthetic polymers are indeed useful biomaterials with customisable and versatile features for TE technologies, but their absence of the inherent biomimicry that is a feature of natural polymers can oppose their universal application.

In order to address the respective shortcomings of both natural and synthetic polymers in TE, composite biomaterials entailing combinations of both have been the subject of many studies, particularly in tissue regeneration applications. Typically, natural and synthetic composites have been designed with the aim of bolstering the stability and robust mechanical properties of synthetic materials with the cell adhesive and cell instructive cues of natural polymers. For example, PCL has been combined with a wide range of natural biomaterials including collagen [77], gelatin [83,84], hyaluronic acid [85], and cellulose [86]. The myriad of all combinations of synthetic-natural composite combinations in the TE field are vast; a comprehensive list is beyond the scope of this mini-review. However, from a structural perspective, the common theme of improved mechanical properties prevails within the literature. As recently illustrated by Jakus and colleagues [87], however, improving structural strength can also yield resultant effects on cell behaviour and the improved development of organotypic tissue. This material, a mix of PCL or PLGA with hydroxyapatite, exhibited high material stiffness, osteogenic differentiation of seeded stem cells, and formed vascularised bone tissue *in vivo*. It well-known in TE that a stiffer material substrate can stimulate stem cell differentiation into bone cells [4], and the presence of the bone mineral hydroxyapatite also contributed to osteogenesis, as with other studies of doping PCL constructs with mineral [83,88]. Once again, the core structural features of the polymers provide a resultant effect on tissue formation as a function of biomaterial composition, mechanical properties, and biodegradation.

## 4 Biophysical Signalling in Tissue Engineering: Cell-Substrate Mechanotransduction

Regardless of whether the biomaterial substrate is natural or synthetic, once cells can attach to a polymer, a combination of receptor-mediated and mechanical-mediated signals will regulate their phenotype and function. This process is defined as mechanotransduction, in which cells sense and respond to mechanical stimuli by converting them to biochemical signals, commencing with cell recognition of specific extracellular motifs to bind, subsequent probing of the physical nature of its surrounding environment, and resultant effector responses [26]. Effector responses to cell ligand density and matrix elasticity include differentiation of stem cells [4,5,89], migration [90,91], and disease progression [[92], [93], [94], [95]].

**Fig. 3:**
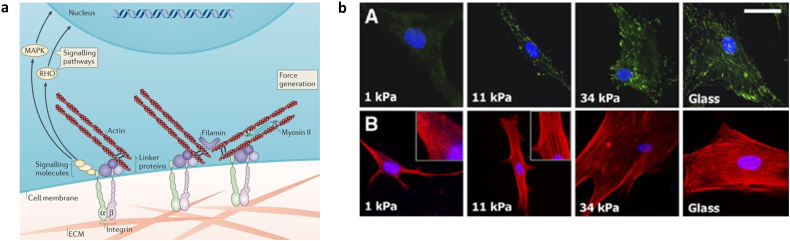
(a) Overview of mechanotransduction. Integrin receptors recognise and bind to cell-adhesive ligands in the extracellular matrix (ECM), initiating the formation of intracellular focal adhesion complexes (left panel). Signalling molecules directly stimulate downstream transcription and linker proteins in the complex bind to actin filaments, which can generate a tensile force in conjunction with myosin II activity. Adapted from [26]. (b) Increased substrate stiffness induces the formation of focal adhesion complexes (green) and actin polymerisation and alignment (red) in human mesenchymal stem cells. Adapted from [4]. (For interpretation of the references to colour in this figure legend, the reader is referred to the web version of this article.)

Mechanotransduction initiates with cell recognition of ligands at the cell-substrate interface, followed by tension generation in the cell and kinase activity, before culminating in downstream signalling responses (Fig. 3a). A fully comprehensive review of all protein-material interactions, including nanotopographical characteristics of the biomaterial and ligand spacing (reviewed in [96]), is beyond the scope of this mini-review but the critical concept of cell responses to adjacent polymeric structures is illustrated through canonical integrin receptor signalling, a family of transmembrane proteins that are the major cell adhesion receptor for the ECM [97,98]. As heterodimeric proteins, distinct α- and β-subunits of integrins can propagate different downstream effects, even upon recognition of the same extracellular ligand. Thus, while integrins are typically classified by their recognition of collagen, fibronectin or laminin motifs, resultant cell responses can range from homeostatic to pathogenic [99].

In either case, extracellular integrin-ligand binding induces conformational changes in the receptor's intracellular domain, stimulating the association of linker proteins including talin [100], vinculin [101], and paxillin [102] to form a focal adhesion complex. The linker proteins in turn facilitate the association of the cytoskeletal actin framework with the focal adhesion complex, generating a tensional force across the cell in proportion to the substrate stiffness sensed in resistance to the net inward flow of actin towards the nucleus. As the stiffness increases, more focal adhesions cluster and actin filaments align to generate a greater tensional force (Fig. 3b). Consequently, the polymerisation of actin filaments and tensional force is felt along the entire cytoskeleton, regulating differential gene expression in response.

In addition to acting as key transducers of mechanical signalling at the cell-substrate interface, focal adhesion subunits, including focal adhesion kinase (FAK), can phosphorylate and activate a range of other pathways with a multitude of effects [103]. In one of its roles, FAK mediates linker protein recruitment as filament tension increases [104], but its kinase activity affects MAPK signalling and Rho kinase activity, among others [[105], [106], [107], [108], [109]]. Herein, the variety of pathways that FAK is linked to reveal its potential for both normal and aberrant signalling in different cells and tissue. For example, FAK knockout in keratinocytes abrogates adequate cell coverage and tissue formation in wound healing [110], while in breast cancer, FAK can operate as a central regulator of invadopodia formation and consequently, matrix proteolysis, cell migration, and metastasis [111]. Thus, via FAK activity, integrin coupling to different biomaterial structures can instigate a complex but coordinated overlap of cellular processes, with a net influence on both essential and unwanted outcomes in our bodies [112]. From a TE perspective, careful biomaterial design can ultimately harness biophysical signalling pathways to elicit the required cell responses in different applications.

Interestingly, biomaterials can also trigger biophysical cell responses in a dynamic fashion through stimuli-responsive polymers. Piezoelectric polymers, for example, develop a voltage in response to a mechanical stress with resultant changes in electroconductive properties and surface charge [113]. Many native tissues are piezoelectric in nature and accordingly, it is little surprise that stem cells respond by differentiating towards myogenic [114], osteogenic [115,116], or chondrogenic [117] lineages. Moreover, electroactive biomaterials have the potential to be combined with external magnetic fields to induce differential cell responses as a function of applied electrical fields [118], potentially enabling “real-time” control of signal transduction. As well electroactive biomaterials, other stimuli-responsive polymers have been explored using pH, temperature, and photo-catalysed reactions [119]. Indeed, in the case of the latter, Ondeck and colleagues have recently shown in an elegant study how stimuli-responsive biomaterials can be used to explore *in situ* cell transformation. Using a methacrylated hyaluronic acid substrate that stiffens under UV light, a mechanosensitive epithelial-mesenchymal transition in precancerous breast epithelial cells was dynamically stimulated, observing an increase in cell invasion as the substrate adopted the mechanical properties of tumour tissue [120]. Ultimately, from a TE perspective, such careful biomaterial design can ultimately harness biophysical signalling pathways, whether regenerative, aberrant, or otherwise to elicit the required cell responses in different applications.

## 5 Summary & Outlook: Convergence of Tissue Engineering With Computational and Structural Biotechnology Applications

The central objective of all TE research is to emulate the anatomical and physiological or pathophysiological traits of a tissue or organ: multicellular systems with accurate spatial distribution of cells and ECM with the appropriate architecture, and resultant coordinated biological responses within this system. Through our knowledge of the structural influence of the 3D scaffold structures on cellular function, our capacity to engineer platforms that repair injury, model disease, and test novel therapeutics has increased significantly. Thus, as the complexity of these platforms increase, so too does the need for advanced methods of analysis that can be found within the realm of computational and structural biotechnology. This outlook serves to highlight recent examples of TE engaging with several methods in this domain to elevate the field to a deeper level of understanding of cellular function in a 3D environment (Table 1).

**Table 1:** Advanced analytical methods from computational and structural biotechnology and their use in analysis of biomaterials. FLIM = Fluorescence-lifetime imaging microscopy; PEG = Polyethylene glycol; PVA = polyvinyl alcohol; RNA-seq = RNA sequencing.

Method	Biomaterial system	Comments	Ref
FLIM	Cellulose-based scaffold	FLIM permitted the real-time acidification of 3D environments by colon cancer cells and stem cell organoids	[121]
	Bovine pericardium tissue	Collagenase-mediated degradation was observable using FLIM	[122]
		Live imaging of recellularisation and vascularisation detectable using FLIM	[123]
	Collagen-based hydrogel	Longitudinal monitoring of collagen crosslinking in real time detectable using LFIM	[124]
RNA-seq	PVA hydrogel	RNA-seq identified enrichment of differentially expressed genes in metabolic activity and cytoskeletal proteins in response to different PVA substrate stiffness	[125]
	Collagen-Matrigel hydrogel	RNA-seq validated the ability of a customised miniature ventricular heart chamber to induce expression of cardiac-specific cellular markers derived from human pluripotent stem cells	[126]
	Silk film	RNA-seq revealed that alignment of silk fibres in films induced differential gene expression in cell adhesion and cytoskeletal dynamics	[127]
	Alginate hydrogel	RNA-seq identified enrichment of differentially expressed genes in cell differentiation and immunomodulatory function as a response to different alginate substrate stiffness	[60]
	PEG hydrogel	RNA-seq validated the ability of the hydrogel biomaterial to induce expression of vascularisation genes in endothelial cells derived from induced pluripotent stem cells	[128]

As a science that develops complex 3D biomimetic structures, advanced imaging capabilities that can combine spatial orientation with functional analysis show great promise for TE platforms. Moreover, the possibility of non-destructive imaging of cells in TE constructs would be clearly of benefit to monitor their *in situ* behaviour in real time. Fluorescence-lifetime imaging microscopy (FLIM) is one such technique [129]. FLIM utilises contrast in live images by spatial variations in fluorescence lifetime of a probe that is largely concentration- and intensity-independent but is sensitive to the environmental surroundings of the fluorophore. In this manner, cell responses including intracellular protein-protein interactions [130] and metabolism [131,132] can be evaluated, with the potential to identify spatially-dependent or other microenvironmental responses. For the TE field, FLIM has only recently began to be recognised as a powerful technique [[121], [122], [123], [124],^133^]; it remains as an exciting technique for further exploitation, with notable possibilities for detailed analysis of integrin agonism [134]. Moreover, interferometric photoactivated localisation microscopy (iPALM) has the capability to examine integrin structures on a nanoscale, offering new insights into differential receptor conformations and related interactions with other biomolecules and signals [[135], [136], [137]]. Finally, as next generation sequencing becomes more commonplace in TE [60], recent work from the Church lab has developed a protocol to combine single cell transcriptomics with spatial orientation [138]; although there are still several technical and logistical hurdles to overcome for widespread use of this technology, it emphasises yet again the potential advanced imaging technologies available to embrace for enhanced analysis of structural interactions in TE systems.

Coupled with advanced imaging techniques for merging structural biotechnology investigations with TE, detailed quantification of the composition of TE platforms can be performed through large-scale analysis with bioinformatics. This is of particular interest as 3D *in vitro* models evolve in conjunction with a deeper understanding of ECM and cell-substrate interactions in disease. Quantitative proteomic analysis of ECM changes in disease, for example, can reveal matrix signatures that could be recapitulated for biomimetic disease models; such studies of the matrisome in cancer and fibrosis are of great interest in this regard [[139], [140], [141]]. RNA sequencing has also begun to feature more in analyses of the biophysical effects of different microenvironments [60,[125], [126], [127], [128]]. Naturally, bioinformatics will have a role to play in the processing of large data related to spatially-relevant transcriptomics [138], and additionally, computational technology will enable intelligent combinatorial analyses of spectroscopic and microscopic data, as has been recently reported in studies that blended histology with Raman spectra and atomic force microscopy to evaluate the mechanical, compositional, and structural characteristics of diseased tissue [142,143].

In summary, the design and development of TE constructs, whether for therapeutic or scientific applications, hinges upon its biomimetic structural features that affect mechanical properties, stability, degradation, cell adhesion, and cell functionality. As TE studies integrate techniques that have been traditionally restricted to computational and structural biologists, greater opportunities for complementary investigations within both disciplines will present themselves. Ultimately, future collaboration provides greater success for both fields; and in order for further advances in a discipline that continues to evolve and address new challenges in the treatment of injury and disease, we propose increased engagement between the interrelated disciplines of structural biotechnology and tissue engineering.

## Conflict of Interest Disclosure

The authors declare no competing financial interest.

## Acknowledgements

The authors acknowledge the funding received for this research by the Government of the State of Kuwait. The graphical abstract was prepared using images available from Servier Medical Art by Servier, licensed under a Creative Commons Attribution 3.0 Unported License (CC BY 3.0).
